# Acute Iridocyclitis Associated With Intravenous Zoledronic Acid: A Case Report

**DOI:** 10.7759/cureus.43162

**Published:** 2023-08-08

**Authors:** Anvitha R Ankireddypalli, Shalamar Sibley

**Affiliations:** 1 Department of Endocrinology, University of Minnesota Medical School, Minneapolis, USA; 2 Endocrinology, Diabetes, and Metabolism, Minneapolis Veteran Affairs Health Care System, Minneapolis, USA

**Keywords:** anterior uveitis, zoledronic acid, bisphosphonates, osteoporosis, ocular inflammation

## Abstract

Bisphosphonates are widely used drugs for the management of osteoporosis. Intravenous (IV) zoledronic acid (ZA) is frequently prescribed in cases of oral bisphosphonate intolerance or non-compliance. Well-known immediate ZA side effects include flu-like symptoms such as nausea, myalgias, bone and joint pains, and fever. Here we report a case of a rare side effect of acute anterior uveitis following initial dosing of ZA in a 71-year-old female with osteoporosis who had been vitamin D deficient a couple of months earlier. She presented with headache, bilateral eye redness, and pain post ZA infusion. Findings of diffuse conjunctival injection, and flare with cells in the anterior chamber were suggestive of anterior uveitis. Her symptoms resolved with prednisolone eye drops in three weeks. Ocular inflammation is a rare but serious side effect of this commonly administered drug. Optimizing vitamin D levels prior to treatment may help to prevent this condition. Clinicians should be aware of the rare occurrence of post-ZA ocular inflammation. Early recognition and prompt treatment are essential.

## Introduction

Bisphosphonates are a commonly used medication in the treatment of osteoporosis. Other indications for bisphosphonates include multiple myeloma, bone metastases, malignancy-induced hypercalcemia, and Paget’s disease [1]. Bisphosphonates are structurally like inorganic pyrophosphates and act by binding to the hydroxyapatite site on the bone undergoing active resorption. Bisphosphonates are absorbed by osteoclasts leading to apoptosis thus suppressing bone resorption [2].

Oral and intravenous formulations are available and vary in their potency as well as adverse effect potential. Intravenous formulations such as zoledronic acid (ZA) are associated with a higher adverse effect rate but are preferred in those intolerant to oral medication or with poor compliance [2]. Documented side effects of bisphosphonates include hypocalcemia, renal toxicity, nausea, anorexia, Barrett’s oesophagus, and atrial fibrillation. An increased incidence of atypical femur fractures and jaw osteonecrosis has been reported as well [3,4].

Well-known immediate ZA side effects include an acute phase reaction (10-30%) with flu-like symptoms such as nausea, myalgia, and fever. The pathophysiology is thought to be through activation of the immune system and induction of an acute phase reaction by stimulating a transient release of proinflammatory cytokines from monocytes, macrophages, and T cells, mainly gamma-delta T cells. The incidence reduces with subsequent doses to 2.8% after the third dose [4,5]. Ocular inflammation is a less well-known side effect of bisphosphonates and is reported in 0.4% [6]. Here, we report a case of a rare side effect of acute anterior uveitis following initial ZA dosing.

## Case presentation

A 71-year-old female was diagnosed with osteoporosis; dual-energy X-ray absorptiometry (DEXA) scan had the lowest T score of -2.6 at the lumbar spine. Vitamin D level was 10 ng/mL. She received calcium and vitamin D supplementation. Two months later, vitamin D level was 31 ng/mL. ZA was recommended due to difficulty with adherence to oral bisphosphonate. Five days after infusion, she presented to our emergency department (ED) with progressive symptoms of bilateral eye itching, redness, excessive tearing, blurry vision, photosensitivity, floaters, pain, and headaches that had begun one day after infusion.

She had two prior outside ED visits for these symptoms and received polymyxin B and later ofloxacin eye drops, which did not relieve them. She denied fever, chills, arthralgia, and myalgia. She denied any prior known autoimmune disorder or prior history of eye symptoms. On examination, she was afebrile and had bilateral conjunctival injection, excess tearing, and watery discharge. She was discharged with olopatadine eye drops and cetirizine, diagnosed with allergic conjunctivitis, and referred to ophthalmology.

On ophthalmic exam, seven days after her infusion, visual acuity was 20/60 in the right eye, and 20/50 in the left eye. Intraocular pressure was 12 mmHg in the right eye and 16 mmHg in the left eye. A slit lamp eye exam showed a diffuse conjunctival injection of the right eye, mild corneal endothelial folds, and flare with cells in the anterior chamber, and synechiae posterior to the iris. Right acute iridocyclitis was diagnosed, and she received prednisolone eye drops and cyclopentolate eye drops. Symptoms improved in one week with almost complete resolution after three weeks of therapy. She has not had a recurrence of these symptoms and has declined future treatment with ZA.

## Discussion

The HORIZON-Pivotal Fracture Trial reported eye pain in 0.2% (zero in controls) and eye inflammation in 0.4% (0.1% in controls) in patients receiving bisphosphonates [6]. As has been documented in several case reports, ocular inflammation following bisphosphonate use can involve almost any part of the eye like proptosis, periorbital edema, extraocular muscle involvement, orbital fat involvement, myositis, scleritis, uveitis, and optic neuritis [7-16]. A cohort analysis by Pazianas et al. suggested that the presence of basal inflammation may predispose to the development of orbital inflammation following bisphosphonates [17]. However, ocular effects have also been reported in patients with suppressed immune responses such as patients being treated for bone metastasis and multiple myeloma [17].

Oral bisphosphonates are more commonly prescribed than intravenous bisphosphonates. Nonetheless, ocular adverse effects are more common in patients receiving intravenous bisphosphonates. This is thought to be due to their higher potency and stimulation of acute phase reactions [18]. The onset of symptoms is within a few hours to 10 days following administration with more rapid onset following intravenous administration. Most occur within three days with intravenous formulations [18,19]. Keren et al. reported that the most common findings were ocular conjunctival hyperemia followed by restriction of ocular movement and proptosis. The most common diagnosis was scleritis. Posterior scleritis can cause pain without other external signs and lead to vision loss. Hence, early diagnosis and treatment are important [20].

Fraunfelder et al. reported a higher prevalence of anterior uveitis with agents given by intravenous route, which was induced mostly by zoledronate and pamidronate, less by alendronate, and never by risedronate [21]. A higher incidence of anterior uveitis has been reported in patients with vitamin D deficiency prior to the start of bisphosphonate therapy. Several studies have established a link between low vitamin D levels and increased activity of uveitis. This adverse effect has been hypothesized to be associated with increased levels of pro-inflammatory cytokines including interleukin (IL)-1, IL-6, and tumor necrosis factor (TNF)-alpha [22]. There is some evidence for higher rates of acute phase reaction in patients with lower levels of vitamin D [22]. Though the current patient’s vitamin D status was adequate at the time of treatment, it had been low two months prior. Thus, we suggest replenishment of vitamin D before starting bisphosphonate therapy [22].

Imaging with ocular ultrasound, CT, and MRI has been used to confirm the presence of ocular inflammation [23]. There have been contradicting reports of recurrence of ocular symptoms following rechallenge. Phillips and Newman [24] and Patel et al. [25] reported no recurrence in patients after rechallenge with zoledronate. Other reports suggest significant recurrence of symptoms following pamidronate [3,21]. It is also unclear if switching medication will reduce adverse effects. There is some evidence to suggest that ocular inflammation is a class effect [16]. Hence, further research is needed to determine if rechallenge with intravenous agents is safe.

## Conclusions

Here we presented a case of anterior uveitis post ZA infusion in a patient with osteoporosis who was previously Vitamin D deficient. ZA-associated ocular inflammation is a rare but serious side effect. This adverse effect has been hypothesized to be associated with increased levels of pro-inflammatory cytokines including IL-1, IL-6, and TNF-alpha. Low vitamin D levels have been associated with an increased incidence of anterior uveitis. Optimizing vitamin D levels prior to bisphosphonate treatment may help to prevent this condition. There is some evidence for higher rates of acute phase reaction in patients with lower levels of vitamin D, but the present patient’s vitamin D status was adequate at the time of treatment. There are conflicting reports of recurrence of symptoms following rechallenge with intravenous bisphosphonates and further research is needed. The decision to rechallenge should be on a case-by-case basis after weighing the risks and benefits. Clinicians should be aware of the rare occurrence of post-ZA ocular inflammation. Early recognition and prompt treatment are essential to prevent vision loss.
